# Recent advances in functional neuroimaging analysis for cognitive neuroscience

**DOI:** 10.1177/2398212817752727

**Published:** 2018-01-19

**Authors:** Nitin Williams, Richard N. Henson

**Affiliations:** 1 Neuroscience Center, Helsinki Institute of Life Science, University of Helsinki, Helsinki, Finland; 2 MRC Cognition and Brain Sciences Unit, University of Cambridge, Cambridge, UK

**Keywords:** Neuroimaging methods, functional magnetic resonance imaging analysis, electro-/magneto-encephalography analysis, multivariate pattern analysis, time-varying connectivity, dynamic connectivity, neurobiological modelling, biophysical modelling, reproducibility, big data

## Abstract

Functional magnetic resonance imaging and electro-/magneto-encephalography are some of the main neuroimaging technologies used by cognitive neuroscientists to study how the brain works. However, the methods for analysing the rich spatial and temporal data they provide are constantly evolving, and these new methods in turn allow new scientific questions to be asked about the brain. In this brief review, we highlight a handful of recent analysis developments that promise to further advance our knowledge about the working of the brain. These include (1) multivariate approaches to decoding the content of brain activity, (2) time-varying approaches to characterising states of brain connectivity, (3) neurobiological modelling of neuroimaging data, and (4) standardisation and big data initiatives.

## Introduction

For many years, the main use of functional neuroimaging has been to localise cognitive processes in space (e.g. with functional magnetic resonance imaging (fMRI)) or time (e.g. with electro-encephalography analysis (EEG)), by comparing brain activity in two or more experimental conditions that differ in the cognitive process of interest (Box 1). More recently, there has been a growing interest in how activity in one brain region relates to activity in other regions, that is, functional connectivity. While these types of analysis have produced impressive advances in knowledge, they tell us little about, for example, the content of brain activity, that is, what that activity might represent in the world or what it might represent to other brain regions.

**Box 1.** Basics of fMRI, EEG, and MEG.

The non-invasive nature of fMRI, EEG, and MEG, as well as their broad coverage of activity in the brain, make them useful to study biological substrates of cognition. While all these methodologies record activity from large populations of neurons, each of them has unique strengths and limitations. fMRI measures the BOLD (blood-oxygen-level-dependent) signal, which reflects the ratio of oxygenated to deoxygenated blood. Since this ratio depends on neuronal population activity, fMRI indirectly measures brain activity. It has a spatial precision of a few millimetres and records activity from all regions in the brain, that is, both cortical and sub-cortical regions. Its ability to provide a window into brain processing during cognition is however limited by its coarse temporal precision – it can only differentiate changes in brain activity occurring around 1 s or more apart. Recent developments like high-field MRI (De Martino et al., 2015) and multi-band fMRI (Todd et al., 2016) promise to further increase the temporal and/or spatial precision offered by fMRI.
Extracranial EEG measures electrical activity from neuronal populations via electrodes on the scalp, specifically post-synaptic potentials of tens of thousands of neurons firing simultaneously. Due to physics of the measurement, EEG activity predominantly reflects post-synaptic potentials of pyramidal neurons near the cortical surface, although sub-cortical contributions are also present, and secondary currents and volume conduction complicate the pattern. EEG can measure neuronal activity with fine temporal detail; this also means that activity in different frequency bands can be resolved with EEG. However, EEG suffers a coarse spatial resolution. This is because of blurring that occurs when electrical fields are propagated through regions of different conductivities (e.g. CSF and scalp) to EEG electrodes, making it difficult to infer location of active brain regions.
MEG measures magnetic induction produced by the post-synaptic electrical activity in neuronal populations measured by EEG. However, due to the different properties of magnetic and electrical fields, the activity recorded by MEG is less affected by secondary currents and more sensitive to superficial sources. At the same time, however, MEG sensors cannot detect the radial component of those currents. Like EEG, MEG can record brain activity with fine temporal and spectral resolution. Crucially though, MEG is less affected by blurring owing to different tissue types. Thus, MEG combines a high temporal resolution with a superior spatial resolution to EEG, in the order of a few centimetres (although even so, localisation is rarely certain, owing to the inverse nature of the mapping from sensors to sources). Recent developments in MEG, that is, optically pumped magnetometers (OPMs) offer the potential for MEG sensors closer to the head, which should further increase signal-to-noise ratios (Boto et al., 2017).

fMRI: functional magnetic resonance imaging; EEG: electro-encephalography; MEG: magneto-encephalography; CSF: cerebrospinal fluid.

### Multivariate pattern analysis

The emphasis on localisation led to the adoption of ‘mass univariate’ analyses, where a separate statistical test (e.g. between two conditions) is done at every point in space or time. By contrast, multivariate analyses use information in the pattern of brain activity over multiple measurements, such as voxels in fMRI, or sensors or time points in electro-/magneto-encephalography (E/MEG). Modern machine learning methods are very powerful at classifying these patterns according to two or more classes, enabling the extraction of small signals from noisy data that would be difficult to detect in univariate analyses (see Haynes, 2015, for primer on pattern analysis approaches). Such ‘decoding’ has been used to track the temporal evolution of cognitive representations using E/MEG. For example, King et al. (2014) trained a classifier on patterns of activity over MEG sensors at one time point and tested its ability to generalise to other time points forward and backward in time. When applied to responses evoked in an auditory mismatch task (where a sequence of expected sounds is followed by unexpected one), these authors were able to characterise an extended sequence of successive processing stages that would be difficult to detect with conventional univariate analyses of evoked responses. These multivariate classifiers can also be used for more conventional localisation, or brain mapping, using ‘searchlight’ methods, where patterns are defined over a subset of spatially or temporally contiguous samples (e.g. a sphere of brain voxels), and these subsets systematically sampled across the data space (e.g. Su et al., 2014 in source-reconstructed E/MEG).

Multivariate patterns can be used for more than simple classification. For example, by measuring the similarity between the patterns evoked by different stimuli, one can construct an *N × N* matrix of all pairwise similarities between a set of *N* stimuli and examine structure in those matrices (e.g. groupings of animate vs inanimate objects) – so-called representational similarity analysis (RSA; Kriegeskorte et al., 2008). Because this representational space is abstracted from the original measurement space, these matrices can be compared across different types of data, for example, human versus nonhuman primate fMRI data, fMRI versus E/MEG data, or even fMRI data versus data simulated by artificial neural networks. In the latter case, RSA has been used to compare patterns in layers of deep-convolutional neural networks of visual categorisation with those in various stages of the human visual object pathway (Khaligh-Razavi and Kriegeskorte, 2014).

More generally, multivariate pattern analysis (MVPA) has enabled questions about mental representations that could not be addressed directly before (e.g. with purely behavioural methods), such as evidence for the suppression of some memories when others are retrieved (Wimber et al., 2015). An interesting future direction of analysis will be multivariate connectivity, where the connectivity between two brain regions is not confined to temporal correlations of univariate signals but rather systematic covariation over time in the patterns of activity in those regions (Geerligs et al., 2016). This has the potential to elucidate how changes in the content of what is represented by one brain region relate to changes in what is represented by another brain region, allowing closer mapping to artificial neural network models of cognitive processing.

Like most analysis techniques, there are pitfalls associated with MVPA. For example, it is vital that the data used for training classifiers are independent of the data used to assess classification performance; otherwise, classification will be biased to be above chance. Furthermore, resulting weight maps of features (e.g. voxels) only reflect how the set of features together achieve classification and cannot be used to make inferences about individual features. Most importantly, like with all neuroimaging data, above-chance classification does not imply that the predictive information is actually used for computation by the brain.

### Time-varying functional connectivity

Traditional measures of functional connectivity assume that networks remain stable across time, for several minutes in the case of resting-state fMRI. This stationarity assumption is normally necessary to obtain sufficient time points to estimate connectivity accurately. While there are attempts to measure time-varying connectivity with fMRI using sliding windows (e.g. Allen et al., 2014), insight is limited by the slow sampling rate (fMRI images are typically only acquired every 1–2 s) and slow dynamics of the haemodynamic response, which obscure changes in brain states at the sub-second scale. The much richer temporal information in E/MEG however allows more advanced methods, such as hidden Markov models (HMMs) (see O’Neill et al., 2017, for a primer on such approaches). Baker et al. (2014), for example, used HMMs to identify a set of states in source-reconstructed resting-state MEG data, whose spatial maps corresponded to the well-known resting-state networks (RSNs) seen in fMRI. However, these RSNs were found to occur transiently, for much shorter durations (100–200 ms) than could be seen with fMRI. Furthermore, the transition probabilities could be examined, with some states being more likely to follow others. An extension of this method was applied to MEG data from a motor task (Vidaurre et al., 2016), revealing a distinct temporal order of states, each associated with frequency-specific motor networks. Notably, some states were time-locked to the task execution, even though no details about task timings were included in the analysis. This type of information about the rapid formation and dissolution of networks during tasks is likely to enrich cognitive neuroscience theories. A current difficulty of these methods is selecting certain parameter values, such as the number of hidden states, which can require considerable computation to optimise.

### Neurobiological modelling

While most existing analysis methods provide statistical descriptions of neuroimaging data, there is a growing interest in understanding the neurobiological mechanisms generating those data. For example, there has been a lot of work on ‘neural mass models’ that approximate the behaviour of populations of neurons and can be used to generate E/MEG and/or fMRI data (Friston et al., 2017). By inverting these generative models, neural-level parameters (e.g. local γ-aminobutyric acid (GABA) concentration) can be inferred from neuroimaging data (Shaw et al., 2017). This allows neuroimaging data to bridge the gap between other levels of neuroscientific investigation, such as single-cell recording data and pharmacological interventions.

There has also been a lot of work on simple neurobiological models that generate RSNs, in particular their graph-theoretic properties, such as small-worldness and presence of hubs (see Cabral et al., 2014, for a primer on different modelling approaches). Deco and Jirsa (2012) for example proposed a whole-brain model of 66 brain regions, whose dynamics were described by integrate-and-fire neurons, convolved with a haemodynamic response to fit fMRI data. By connecting these regions in accordance with known anatomical connectivity (from diffusion MRI tractography), RSNs and their graph properties were observed to emerge from the model as noise-induced fluctuations around a stable low activity state. This work has been extended to RSNs in resting-state MEG. Nakagawa et al. (2014) used the same model as Deco and Jirsa (2012) to show that realistic conduction delays are important to reproducing MEG RSNs. More specifically, they demonstrated that correlations between alpha-band power envelopes were most similar to those in experimental data when propagation delays along white-matter tracts were within the known physiological range (5–10 m/s). These network models can be particularly helpful in simulating brain development and brain changes associated with disease. Nevertheless, it should be noted that these modelling efforts are at an early stage. While current models can emulate some of the phenomena observed (e.g. graph properties), there are many other phenomena (e.g. transient responses and travelling waves) that they do not yet capture.

### Big data, standardisation, and reproducibility

Like other fields of science, neuroimaging has suffered from failures to reproduce results (see Poldrack et al., 2017 for a primer on issues relating to reproducibility). Part of the reason is the many degrees of freedom in analysis choices, which can inadvertently lead to false positives owing to failure to correct for the multiple analyses performed. This is being actively addressed with attempts to standardise analysis pipelines (e.g. https://www.nitrc.org/), ideally in advance of data collection. This convergence towards standard practice (e.g. Gross et al., 2013; Poldrack et al., 2008) is typical in relatively young fields like neuroimaging (which only really took-off in the 1990s).

Another problem has been the small sample sizes in many imaging studies (Ioannidis, 2005), producing low power, which interacts with the well-known publication bias for reporting positive results. This problem is being addressed by meta-analyses and initiatives to pool data (mega-analyses), again helped by standardisation, in this case of data formats (e.g. http://bids.neuroimaging.io/). More and more large datasets are being made available to other researchers (e.g. Human Connectome Project, http://www.humanconnectomeproject.org/; Alzheimer Disease Neuroimaging Initiative, http://adni.loni.usc.edu/), as part of the growing open science movement. Many of these datasets include multiple neuroimaging modalities, requiring development of methods for multi-modal integration (e.g. Henson et al., 2011), with the goal of combining the spatial resolution of fMRI with temporal resolution of M/EEG. A challenge for the future will be to encourage standardisation without stifling future development of new methods.

## Conclusion

The rapid advances in analysis methods for fMRI and E/MEG data make it an exciting time for cognitive neuroscience, as we hope some of the examples above illustrate. While MVPA and time-varying functional connectivity allow fundamentally new questions to be asked, neurobiological modelling offers a means to a deeper understanding of mechanisms generating the data. These approaches are complemented by the improved reproducibility of results that accompanies standardisation and use of big data. We look forward to the continued expansion, optimisation, and standardisation of the neuroscientist’s neuroimaging toolkit.

## References

[bibr1-2398212817752727] Allen EA Damaraju E Plis SM, et al (2014) Tracking whole-brain connectivity dynamics in the resting-state. Cerebral Cortex 24(3): 663–676. 23146964 10.1093/cercor/bhs352 PMC3920766

[bibr2-2398212817752727] Baker AP Brookes MJ Rezek IA, et al (2014) Fast transient networks in spontaneous human brain activity. eLife 3: e01867. 24668169 10.7554/eLife.01867 PMC3965210

[bibr3-2398212817752727] Boto E Meyer S Shah V, et al (2017) A new generation of magnetoencephalography: Room temperature measurements using optically-pumped magnetometers. NeuroImage 149: 404–414. 28131890 10.1016/j.neuroimage.2017.01.034 PMC5562927

[bibr4-2398212817752727] Cabral J Kringelbach ML Deco G (2014) Exploring the network dynamics underlying brain activity during rest. Progress in Neurobiology 114: 102–131. 24389385 10.1016/j.pneurobio.2013.12.005

[bibr5-2398212817752727] De Martino F Moerel M Ugurbil K, et al (2015) Frequency preference and attention effects across cortical depths in the human primary auditory cortex. Proceedings of the National Academy of Sciences of the United States of America 112(52): 16036–16041. 26668397 10.1073/pnas.1507552112 PMC4702984

[bibr6-2398212817752727] Deco G Jirsa V (2012) Ongoing cortical activity at rest: Criticality, multistability, and ghost attractors. Journal of Neuroscience 32(10): 3366–3375. 22399758 10.1523/JNEUROSCI.2523-11.2012 PMC6621046

[bibr7-2398212817752727] Friston KJ Preller KH Mathys C, et al (2017) Dynamic causal modelling revisited. NeuroImage. Epub ahead of print 17 February. DOI: 10.1016/j.neuroimage.2017.02.045. PMC6693530 28219774

[bibr8-2398212817752727] Geerligs L Cam-CAN Henson RN (2016) Functional connectivity and structural covariance between regions of interest can be measured more accurately using multivariate distance correlation. NeuroImage 135: 16–31. 27114055 10.1016/j.neuroimage.2016.04.047 PMC4922835

[bibr9-2398212817752727] Gross J Baillet S Barnes GR, et al (2013) Good practice for conducting and reporting MEG research. NeuroImage 65: 349–363. 23046981 10.1016/j.neuroimage.2012.10.001 PMC3925794

[bibr10-2398212817752727] Haynes J-D (2015) A primer on pattern-based approaches to fMRI: Principles, pitfalls, and perspectives. Neuron 87(2): 257–270. 26182413 10.1016/j.neuron.2015.05.025

[bibr11-2398212817752727] Henson RN Wakeman DG Litvak V, et al (2011) A parametric empirical Bayesian framework for the EEG/MEG inverse problem: Generative models for multi-subject and multi-modal integration. Frontiers in Human Neuroscience 5: 76. 21904527 10.3389/fnhum.2011.00076 PMC3160752

[bibr12-2398212817752727] Ioannidis JPA (2005) Why most published research findings are false. PLoS Medicine 2(8): e124. 16060722 10.1371/journal.pmed.0020124 PMC1182327

[bibr13-2398212817752727] Khaligh-Razavi S-M Kriegeskorte N (2014) Deep supervised, but not unsupervised, models may explain IT cortical representation. PLoS Computational Biology 10(11): e1003915. 25375136 10.1371/journal.pcbi.1003915 PMC4222664

[bibr14-2398212817752727] King J-R Gramfort A Schurger A, et al (2014) Two distinct dynamic modes subtend the detection of unexpected sounds. PLoS ONE 9(1): e85791. 24475052 10.1371/journal.pone.0085791 PMC3903480

[bibr15-2398212817752727] Kriegeskorte N Mur M Bandettini P (2008) Representational similarity analysis: Connecting the branches of systems neuroscience. Frontiers in Systems Neuroscience 2: 4. 19104670 10.3389/neuro.06.004.2008 PMC2605405

[bibr16-2398212817752727] Nakagawa TT Woolrich M Luckhoo H, et al (2014) How delays matter in an oscillatory whole-brain spiking-neuron network model for MEG alpha-rhythms at rest. NeuroImage 87: 383–394. 24246492 10.1016/j.neuroimage.2013.11.009

[bibr17-2398212817752727] O’Neill GC Tewarie P Vidaurre D, et al (2017) Dynamics of large-scale electrophysiological networks: A technical review. NeuroImage. Epub ahead of print 4 October. DOI: 10.1016/j.neuroimage.2017.10.003. 28988134

[bibr18-2398212817752727] Poldrack RA Baker C Durnez J, et al (2017) Scanning the horizon: Towards transparent and reproducible neuroimaging research. Nature Reviews Neuroscience 18(2): 115–126. 28053326 10.1038/nrn.2016.167 PMC6910649

[bibr19-2398212817752727] Poldrack RA Fletcher PC Henson RN, et al (2008) Guidelines for reporting an fMRI study. NeuroImage 40(2): 409–414. 18191585 10.1016/j.neuroimage.2007.11.048 PMC2287206

[bibr20-2398212817752727] Shaw AD Moran RJ Muthukumaraswamy SD, et al (2017) Neurophysiologically-informed markers of individual variability and pharmacological manipulation of human cortical gamma. NeuroImage 161: 19–31. 28807873 10.1016/j.neuroimage.2017.08.034 PMC5692925

[bibr21-2398212817752727] Su L Zulfiqar I Jamshed F, et al (2014) Mapping tonotopic organization in human temporal cortex: Representational similarity analysis in EMEG source space. Frontiers in Neuroscience 8: 368. 25429257 10.3389/fnins.2014.00368 PMC4228977

[bibr22-2398212817752727] Todd N Moeller S Auerbach E, et al (2016) Evaluation of 2D multiband EPI imaging for high-resolution, whole-brain, task-based fMRI studies at 3T: Sensitivity and slice leakage artifacts. NeuroImage 124(Pt A): 32–42. 26341029 10.1016/j.neuroimage.2015.08.056 PMC4655914

[bibr23-2398212817752727] Vidaurre D Quinn AJ Baker AP, et al (2016) Spectrally resolved fast transient brain states in electrophysiological data. NeuroImage 126: 81–95. 26631815 10.1016/j.neuroimage.2015.11.047 PMC4739513

[bibr24-2398212817752727] Wimber M Alink A Charest I, et al (2015) Retrieval induces adaptive forgetting of competing memories via cortical pattern suppression. Nature Neuroscience 18(4): 582–589. 25774450 10.1038/nn.3973 PMC4394359

